# How hungry roots get their microbes

**DOI:** 10.7554/eLife.82391

**Published:** 2022-09-13

**Authors:** Maggie R Wagner

**Affiliations:** 1 Department of Ecology and Evolutionary Biology, Kansas Biological Survey and Center for Ecological Research, University of Kansas Lawrence United States

**Keywords:** plant microbiome, rhizobiome, GWAS, host-microbe interaction, population genetics, Maize

## Abstract

Maize genes influence which species of bacteria are recruited from the soil, especially in the absence of nitrogen supplied by fertilizer.

**Related research article** Meier MA, Xu G, Lopez-Guerrero MG, Li G, Smith C, Sigmon B, Herr JR, Alfano JR, Ge Y, Schnable JC, Yang J. 2022. Association analyses of host genetics, root-colonizing microbes, and plant phenotypes under different nitrogen conditions in maize. *eLife* **11**:e75790. doi: 10.7554/eLife.75790.

What if farmers could tend to their crops using microbes that naturally occur in the soil rather than expensive and polluting chemicals? Like humans, plants rely on complex microbiomes that contain thousands of different bacterial and fungal species. Many of these microorganisms are highly beneficial to their host: for example, some can break down toxic substances, neutralize disease-causing organisms, increase the availability of water and nutrients, or even boost growth by modifying the balance of hormones in the plant (Busby et al., 2016; Glick, 2012).

Harnessing the power of these microbes for agriculture, however, is easier said than done. Microbiomes are challenging to manipulate due to their complexity, and beneficial species introduced artificially are often outcompeted by countless other soil inhabitants. Rather than attempting to add new organisms to an already crowded system, some researchers are focusing instead on a new target: the genome of the host.

This fascinating approach relies on the fact that the genetic makeup of an individual plant can influence the species of microbes it recruits – a phenomenon known as ‘microbiome heritability’ (Walters et al., 2018). Genetically improving crops so they can attract a superior microbiome from any soil could therefore be possible (Song et al., 2021). Now, in eLife, Jinliang Yang, James Schnable and colleagues at the University of Nebraska–Lincoln — including Michael Meier as first author — report the results of a study on maize (also known as corn) that bring us several steps closer to achieving that vision (Meier et al., 2022).

The team scanned the genomes of individual plants with various genetic backgrounds while also using DNA analysis approaches to examine the composition of their root-associated microbiome (or rhizobiome). First, the analyses revealed dozens of microbial groups which were linked to genetic variations in over 600 regions in the maize genome; crucially, these were also associated with physical traits in the plant. For instance, the abundances of 62 microbial groups were correlated with changes in canopy cover, which is used often as a proxy for plant vigor.

Most of these groups showed evidence of being controlled by the plant’s genotype, suggesting that maize can influence the abundance of soil-dwelling microbes that affect its health. Yet these findings are based on correlations, precluding any definitive conclusions about whether the microbes impact canopy cover or vice versa. While microbial groups could indeed boost leaf growth, it is also possible that plants with denser canopies have higher photosynthesis rates and can therefore produce more sugar to feed mutualistic microbes (Sasse et al., 2018). Still, the results suggest that these heritable microbes are linked to the physical traits of the plants, whether directly or indirectly.

Second, Meier et al. studied with unprecedented detail the regions of the maize genome associated with the composition of the rhizobiome. By combining microbiome data with genome sequence information from 230 maize genotypes, the researchers were able to identify 119 genomic ‘hotspots’ linked to bacterial abundances. Genes within these regions were more likely to be expressed in roots, making them promising candidates for further investigation into the genetic control of the rhizobiome.

In addition, the team gathered evidence that these hotspots may be under purifying selection, an evolutionary process that conserves beneficial genetic variants by eliminating less advantageous ones. As these bacterial groups are associated with canopy cover, this suggests that selection may have been acting on heritable microbes themselves. Careful experimentation is now required to prove that it is in fact the microbes which alter plant fitness and are targeted by natural selection, rather than specific root traits which are important for plant fitness but also happen to be associated with the presence of these microorganisms (MacColl, 2011).

Finally, the work of Meier et al. illustrates how the environment influences the relationship between host genome and microbiome. In their study, plants with identical genetic backgrounds were grown either in fertilized (high-nitrogen) or unfertilized (low-nitrogen) fields. Out of all the bacterial groups examined, only 23% were linked to plant genotype in both conditions; 30% were heritable only in one treatment or the other. In addition, every genomic hotspot was specific to either fertilized or unfertilized fields. It is well known that the environment interacts with genotype to shape phenotype (Des Marais et al., 2013). However, these latest results clearly demonstrate that plants recruit different microbiomes depending on their nutritional needs (Figure 1).

**Figure 1.**
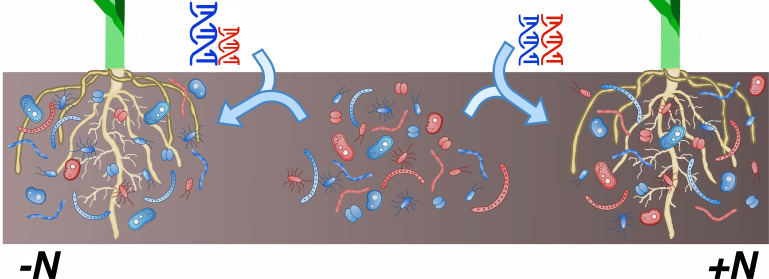
In maize, the composition of the rhizobiome is influenced by interactions between genotype and environment. The rhizobiome of a maize cultivar – the complex community of bacteria and fungi on and near its roots – is recruited from the collection of microorganisms present in the soil (center; red and blue shapes). Differences in genotypes (represented by the DNA helices) between plants influence which microbial species are enlisted to the microbiome. Meier et al. have now shown that certain genetic variants in the host plant affect the relative abundance of microbial groups only in specific environments. For instance, variations at certain loci (blue and red helices) favor the recruitment of specific microbial groups in low-nitrogen conditions (left; taller blue helix and greater number of blue microbial species near the roots) but have no impact in high-nitrogen habitats (right).

These insights into the mechanisms that shape rhizobiome composition also provide an interesting perspective on the evolutionary history of maize. For most of its existence, this species was a wild grass called teosinte, growing in what is now southwestern Mexico (Sánchez González et al., 2018). Over millions of years, natural selection likely favored genetic changes that enabled teosinte to acquire more nitrogen – including those which acted by promoting symbiotic relationships with beneficial microbes (Van Deynze et al., 2018). When growers domesticated teosinte about 9,000 years ago, they probably continued to select these nitrogen-enhancing variants as they picked the most productive genotypes for their fields.

The rise of industrial agriculture in the last century may have changed these evolutionary pressures. The breeding of high-yielding cultivars has coincided with the widespread use of chemical fertilisers. With ample synthetic nitrogen available, the genetic variants that allowed teosinte and earlier maize to acquire nitrogen through root traits and microbial interactions likely became less beneficial (Petipas et al., 2020). In turn, this would have relaxed the purifying selection that had maintained them for millennia, a change which has already been observed in other crops such as legumes (Kiers et al., 2007).

Modern farming practices currently impose a high cost on the environment, challenging us to find ways to make agriculture more sustainable. Beneficial soil-dwelling microbes could be a powerful tool in this effort. Looking forward, knowing which plant genes support these microbes may help us to genetically improve the crops that feed our world.
